# Reports of Scottish Asylums

**Published:** 1918-03-23

**Authors:** 


### The Hospital

The Workers' Newspaper of Administrative Medicine and Institutional Life, Administration, National Insurance and Health.

No. 1659, Vol. LXIII. SATURDAY, MARCH 23, 1918. PRICE ONE PENNY

### REPORTS OF SCOTTISH ASYLUMS.

In our issue of February 23, page 447, we published a letter from Dr. T. C. Mackenzie, Medical Superintendent of the District Asylums, Inverness, in which he took up the cudgels on behalf of Scottish alienists in regard to their use of the annual Reports of asylums as a means of educating the public on the problems of madness. We have now received from Dr. T. C. Mackenzie a number of his annual Reports, and one or two Reports of other institutions for the insane in Scotland, and we are bound, and also pleased, to say that, as far as these institutions are concerned, our reproach that the medical directors of asylums do not take advantage of their opportunities of educating the public with respect to insanity are unfounded. Dr. Mackenzie's own Reports contain surveys that are models of what such surveys should be of the general problems connected with insanity, and we are indebted to him for drawing our attention to them. It is much to be desired that his English colleagues should follow so good an example.

In his Report for 1911 Dr. Mackenzie discusses the widely prevalent opinion that insanity is increasing amongst us, and shows that the increase in the number of admissions into asylums, and in the number of insane persons resident in asylums, does not necessarily mean that insanity is increasing. This had been shown before, but it is just the kind of topic that should be discussed by the managers of asylums in their annual Reports, which reach many people who would not otherwise become acquainted with the facts and their bearing. Moreover, Dr. Mackenzie makes a point that we do not remember to have seen mentioned before, viz., that the standard of insanity has been raised. We are more thorough in our investigations and more exacting in our requirements than we used to be, and many a person whose insanity is not very pronounced, and who would have been passed over in former years as passably competent to manage his affairs, is now taken charge of and committed to an asylum. Whether this is wholly desirable in the cases of elderly persons may well be debated, but Dr. Mackenzie is certainly right in drawing attention to it as a factor in increasing the number of the insane officially recognised. His Report for 1914 discusses the effect of the outbreak of war upon the production of insanity and upon legislation, and makes a laudable endeavour to combat the notion that an opprobrium and a stigma cling to the unfortunate whose disease happens to affect his brain rather than any other organ in his body. It is this cruel and pernicious superstition, to which we have repeatedly called attention, that medical men in charge of asylums can do so much to eradicate, and we welcome Dr. Mackenzie's efforts in this direction. In his Report for the year 1916 Dr. Mackenzie again discusses the problematic increase of insanity, this time in connection with the effects of the war, and he courageously tackles the thorny subjects of alcoholism and venereal disease in connection with legislation. His remarks appear to us sound and sensible, and we have much pleasure in declaring that, as far as he is concerned, our reproach against the superintendents of lunatic asylums, that they neglect their opportunities of educating the public, are unfounded. They are still true, however, of the majority of superintendents, though we admit that the exceptions are more numerous in Scotland than they are in the South. Dr. George Robertson, who has succeeded Sir T. Clouston in the management of Morningside, for which Sir T. Clouston established a European reputation, also discusses in a popular and interesting manner, well calculated to catch the attention of the general public and impress them, various problems of the treatment, causation, and prevention of insanity; and Dr. Easterbrook, of the Crichton Royal Institution at Dumfries, gives, in his Report for the year 1910, a very interesting description of the Continental clinics for insanity, and shows how radically different they are from the lunatic asylums of this country—different, but not necessarily superior. In spite of the extravagant laudation that it was the fashion before the war to bestow upon everything German, but especially upon the German teaching in mental diseases, Dr. Easterbrook, writing four years before the war, showed that we have nothing to learn from Germany in this matter.

We welcome this opportunity of making amends to our Scottish *confrères*, and we are obliged to Dr. T. C. Mackenzie for furnishing us with the materials that enable us to do so.

